# Comparison of central laboratory assessments of ER, PR, HER2, and Ki67 by IHC/FISH and the corresponding mRNAs (*ESR1, PGR, ERBB2*, and *MKi67*) by RT-qPCR on an automated, broadly deployed diagnostic platform

**DOI:** 10.1007/s10549-018-4889-5

**Published:** 2018-08-17

**Authors:** Natalie C. Wu, Wendy Wong, Kenneth E. Ho, Victor C. Chu, Annaliza Rizo, Simon Davenport, Devon Kelly, Rosemary Makar, Jacek Jassem, Renata Duchnowska, Wojciech Biernat, Barbara Radecka, Tomoyuki Fujita, Jonathan L. Klein, Mark Stonecypher, Shoichiro Ohta, Hartmut Juhl, Jodi M. Weidler, Michael Bates, Michael F. Press

**Affiliations:** 1 Division of Oncology Research and Development, Cepheid, Sunnyvale, CA USA; 2 Department of Pathology, Keck School of Medicine, USC/Norris Comprehensive Cancer Center, University of Southern California, 1441 Eastlake Avenue, STE. 5409, Los Angeles, CA 90033-0800 USA; 3 Oregon Health and Science University/Knight Cancer Institute, Portland, OR USA; 4 Medical University of Gdansk, Gdansk, Poland; 5 Military Institute of Medicine, Warsaw, Poland; 6 Oncology Center, Opole, Poland; 7 Tokyo Medical University Ibaraki Medical Center, Ami, Ibaraki Japan; 8 Geneuity/MPLN, Maryville, TN USA; 9 Department of Human Life Science, School of Nursing, Josai University, Sakado, Japan; 10 Indivumed, Hamburg, Germany; 11 Medical and Scientific Affairs and Strategy, Oncology, Cepheid, Sunnyvale, CA USA

**Keywords:** Breast cancer biomarker assays, STRAT4, Estrogen receptor, Progesterone receptor, Human epidermal growth factor receptor 2, Tumor proliferation rate, IHC, FISH

## Abstract

**Purpose:**

The methods (IHC/FISH) typically used to assess ER, PR, HER2, and Ki67 in FFPE specimens from breast cancer patients are difficult to set up, perform, and standardize for use in low and middle-income countries. Use of an automated diagnostic platform (GeneXpert®) and assay (Xpert® Breast Cancer STRAT4) that employs RT-qPCR to quantitate *ESR1, PGR, ERBB2*, and *MKi67* mRNAs from formalin-fixed, paraffin-embedded (FFPE) tissues facilitates analyses in less than 3 h. This study compares breast cancer biomarker analyses using an RT-qPCR-based platform with analyses using standard IHC and FISH for assessment of the same biomarkers.

**Methods:**

FFPE tissue sections from 523 patients were sent to a College of American Pathologists-certified central reference laboratory to evaluate concordance between IHC/FISH and STRAT4 using the laboratory’s standard of care methods. A subset of 155 FFPE specimens was tested for concordance with STRAT4 using different IHC antibodies and scoring methods.

**Results:**

Concordance between STRAT4 and IHC was 97.8% for *ESR1*, 90.4% for *PGR*, 93.3% for *ERBB2* (IHC/FISH for HER2), and 78.6% for *MKi67*. Receiver operating characteristic curve (ROC) area under the curve (AUC) values of 0.99, 0.95, 0.99, and 0.85 were generated for *ESR1, PGR, ERBB2*, and *MKi67*, respectively. Minor variabilities were observed depending on the IHC antibody comparator used.

**Conclusion:**

Evaluation of breast cancer biomarker status by STRAT4 was highly concordant with central IHC/FISH in this blinded, retrospectively analyzed collection of samples. STRAT4 may provide a means to cost-effectively generate standardized diagnostic results for breast cancer patients in low- and middle-income countries.

**Electronic supplementary material:**

The online version of this article (10.1007/s10549-018-4889-5) contains supplementary material, which is available to authorized users.

## Introduction

Breast cancer is becoming increasingly recognized as a major health problem in low- and middle-income countries (LMIC) [1–5]. Although the impact of cancer diagnoses overall has often been overshadowed in these settings by infectious diseases like tuberculosis, malaria, and HIV, the numbers of patients affected by breast cancer is already substantial, and is likely to increase among LMIC in regions where populations are growing the fastest [6].

Currently, however, the treatment of breast cancer in LMIC is fraught with difficulty. In recent years, effective treatments like tamoxifen have become available at low or no cost for women with estrogen receptor (ER) positive breast cancer, accounting for approximately two-thirds of cases. Unfortunately, access to high-quality diagnostic technologies capable of identifying a tumor as ER-positive have been difficult to set up and maintain in a standardized and cost-effective manner (personal communication, John Flanigan, Senior Advisor, Center for Global Health, National Cancer Institute), owing largely to their reliance on antibody-based methods requiring significant expertise to perform and interpret. As lower cost biosimilars of trastuzumab become available [7, 8], breast cancer patients with tumors that overexpress the human epidermal growth factor receptor 2 (HER-2) may find themselves struggling to access a highly effective drug because diagnostic tests that are standard of care for every breast cancer patient in the United States and Europe are unavailable to women in LMIC.

Stimulated by several studies that showed an association between quantitative measurements of mRNA for the transcripts encoding ER and HER-2 (*ESR1* and *ERBB2*) and clinical outcomes on tamoxifen and trastuzumab, respectively [9–11], we anticipated that an assay based on quantitative, real-time, polymerase chain reaction (RT-qPCR) methodology would be highly concordant with central measurements of ER and HER-2 using IHC and/or FISH, and might, therefore, be extremely useful in LMIC. A critical consideration in the genesis of this idea was the fact that such RT-qPCR assays could be developed and run on a distributed diagnostic platform called the GeneXpert®, [(Cepheid, Sunnyvale, CA, USA), (http://www.cepheid.com/us/cepheid-solutions/systems/genexpert-systems/genexpert-i)], which performs automated sample preparation and multiplexed RT-qPCR assays in approximately 2 h. The platform is designed for ease of use, and is already widely distributed throughout the world with more than 17,000 instruments running in 182 countries. Moreover, the platform has been adapted to extract nucleic acids from formalin-fixed, paraffin-embedded tissue (FFPE), the most common tissue-type employed by pathologists for the analysis of breast cancer specimens.

Thus, we aimed to demonstrate that the measurement of mRNAs for the analytes *ESR1* and *ERBB2* were concordant with high-quality central laboratory assessments by immunohistochemistry (IHC) for ER and HER2 protein expression and fluorescence*-*in-situ-hybridization (FISH) for *HER-2* gene amplification. A multiplexed assay was built that included, in addition to *ESR1* and *ERBB2*, primers and probes to detect and quantitate mRNAs for the progesterone receptor (PR, *PGR*) and the cell proliferative antigen identified by monoclonal antibody Ki-67 (Ki67, *MKi67*). The panel is referred to as Xpert® Breast Cancer STRAT4 (STRAT4). Once constructed, the assay was analytically validated by demonstrating linearity and dynamic range, analytical sensitivity (minimal sample input), analytical specificity (tests for interfering substances), prevention of carryover contamination, and assay kit stability (Chu et. al. manuscript submitted for publication). Additional studies were performed to examine the impact of pre-analytical sample handling (selection of invasive carcinoma for testing, macro-dissection techniques, and STRAT4 assay performance by different pathologists) on assay result variability, as well as assess concordance with central IHC/FISH and define preliminary cutoff values (Wong et al. manuscript submitted for publication).

The current study is designed to investigate concordance between STRAT4 and standard IHC and FISH performed at a central laboratory using a large cohort of FFPE specimens tested in a blinded, retrospective manner, and interpreted according to the 2013–2014 ASCO/CAP guidelines. In addition, we examined the concordance between STRAT4 and several different antibodies commonly used in IHC assays performed at different central laboratories.

STRAT4 is a CE-IVD (Conformité Européene In-vitro Medical Device) product that is available in some, but not all, European countries, and is not available in the United States. Where the STRAT4 assay is not available under CE-IVD, evaluations of its performance using specimens prepared under local pre-analytical sample handling procedures can be supported under collaborative research agreements using a Research Use Only version.

## Materials and methods

### Specimen collection, IHC and HER-2 FISH analysis

523 surgically excised breast tumors prepared as FFPE specimens, ranging from 6 months to 22 years in block age, were sourced from five institutes worldwide. Tumor blocks were selected based on what was available at each site. For each specimen, one sectioned slide was stained with hematoxylin and eosin (H&E) and used by pathologists to mark tumor areas, estimate tumor size, and estimate percentage tumor content. Serial unstained tumor samples (4 µm in thickness) were delivered to Cepheid for STRAT4 testing and to the University of Southern California (USC, Los Angeles, CA) Breast Cancer Analysis Laboratory for ER, PR, HER2, and Ki67 IHC and HER-2 FISH analyses. More sections were cut from a subset of tumor blocks (155 out of 523 total blocks) and sent to USC, Molecular Pathology Laboratory Network, Inc. (MPLN, Maryville, TN), and LabCorp (previously Pathology Inc., Torrance, CA) where different antibodies and scoring methods were used to generate IHC results for each analyte. Each reference laboratory generated its own H&E slides for each sample. All IHC, HER-2 FISH and STRAT4 testing was performed within two weeks after block sectioning. Only a subset of samples was tested for concordance for Ki67/*MKi67*. The immunohistochemical assay methods used in the USC central laboratory for assessment of ER, PR, Ki67 and HER2 are described elsewhere [21–24].

### Sample processing and testing for STRAT4

FFPE samples were processed according to the package insert instructions of the STRAT4 assay kit. For each specimen, one unstained slide was overlaid onto the H&E slide which had been marked by a pathologist to select the invasive carcinoma, and then was used to choose the material to be macro-dissected into a 1.5 mL eppendorf tube using a razor blade. Macro-dissected tumor material was then mixed with 1.2 mL of FFPE lysis reagent and 20 µL of proteinase K. The tubes containing the sample lysate were placed in heat blocks for incubation at 80 °C for 30 min. Sample lysate was then mixed with 1.2 mL of ethanol (molecular biology grade, Sigma-Aldrich). For each sample, 520 µL of the lysate was transferred to the sample chamber of a STRAT4 cartridge and placed into a GeneXpert module for RNA extraction, purification, and RT-qPCR analysis.

### GeneXpert DX software analysis settings

*ESR1, PGR, ERBB2*, and *MKi67* mRNA measurements were normalized against the mRNA measurement of the internal reference target Cytoplasmic FMR1-Interacting Protein 1 (*CYFIP1*). Optical readouts of PCR amplifications and cycle threshold (Ct) determination for all targets and *CYFIP1* in STRAT4 test runs were analyzed with settings specified in the GeneXpert DX software. Delta *C*_t_ $\left( {{\text{d}}{C_{\text{t}}}=[{C_{{{\text{t}}_{CYFIP1}}}}] - [{C_{{{\text{t}}_{{\text{target}}\,{\text{gene}}}}}}]} \right)$ assay cutoffs for *ESR1* and *ERBB2* were set at “− 1” and d*C*_t_ cutoffs for *PGR* and *MKi67* were set at “− 3.5” and “− 4”, respectively. Preliminary assay cutoffs were determined in a previous study involving 32 FFPE breast cancer samples that were tested by both STRAT4 and central IHC/FISH in a reference laboratory. The delta *C*_t_ numerical limits were set to maximize the concordance with IHC (IHC/FISH for HER2). To minimize the rate of false negatives for *PGR* and *MKi67*, a minimum assay input value of 31 for the *CYFIP C*_t_ was implemented. If the d*C*_t_ for *PGR* or *MKi67* was lower than the pre-specified cutoffs (d*C*_t_ = − 3.5 for *PGR* or − 4 for *MKi67*) and the *CYIFIP* Ct was greater than 31, the result was reported as “INDETERMINATE” instead of “NEGATIVE” indicating that the minimum assay input criteria had not been met (*CYFIP1 C*_t_ ≥ 31), and the test should be repeated adding more lysate to the cartridge to achieve a *CYFIP1 C*_t_ of at least 31.

## Results

Among 523 specimens tested with the STRAT4 assay, 503 samples yielded valid test results (“POSITIVE” or “NEGATIVE”) for at least one assay target. 20 specimens had no or insufficient PCR amplification signal for the reference RNA *CYFIP1* (*CYFIP1 C*_t_ > 35). Most of these 20 specimens came from FFPE blocks that were more than 10-years old (data not shown).

### Agreement rates between *ESR1* mRNA and ER protein by IHC

The overall concordance rate of the STRAT4 *ESR1* results compared with central IHC results was 97.8% using either a 1% or 10% immunostaining level for positivity (Table 1).

**Table 1:** Comparison of mRNA and protein status for *ESR1* (ER), *PGR* (PR), *ERBB2* (HER2), and *MKI67* (Ki67) determined by IHC and RT-qPCR

Analyte	IHC+/RTqPCR+	IHC−/RTqPCR+	IHC+/RTqPCR−	IHC−/RTqPCR−	Total number	Sensitivity (PPA) (95% CI)	Specificity (NPA) (95% CI)	Kappa statistic (95% CI)	Concordance rate (OPA) (95% CI)
*ESR1* (ER)*	407	4	7	75	493	98.3% (96.5–99.3%)	94.9% (87.5–98.6%)	91.8% (87.1–96.6%)	97.8% (96.0–98.9%)
*ESR1* (ER)^x^	404	7	4	78	493	99.0% (97.5–99.7%)	91.8% (83.8–96.9%)	92.1% (87.4–96.7%)	97.8% (96.0–98.9%)
*PGR* (PR)*	333	23	23	99	478	93.5% (90.5–95.9%)	81.1% (73.1–87.7%)	74.7% (67.8–81.6%)	90.4% (87.4–92.9%)
*PGR* (PR)^x^	320	36	7	115	478	97.9% (95.6–99.1%)	76.2% (68.8–82.7%)	78.1% (71.9–84.2%)	91.0% (88.1–93.4%)
*ERBB2* (HER2)**	66	29	4	391	490	94.3% (86.0–98.4%)	93.1% (90.2% − 95.3%)	76.1% (68.4–83.8%)	93.3% (90.7–95.3%)
*ERBB2* (HER2)^xx^	42	16	2	361	421	95.5% (84.5–99.4%)	95.8% (93.2% − 97.6%)	80.0% (71.1–88.9%)	95.7% (90.7–95.3%)
*ERBB2* (HER2)^xxx^	56	23	6	242	327	90.3% (80.1–96.4%)	91.3% (87.3% − 94.4%)	73.9% (65.0–82.8%)	91.1% (87.5–94.0%)
*ERBB2* (HER2)*** in ER+	39	28	1	341	389	97.5% (86.8–99.9%)	92.4% (89.2–94.9%)	69.1% (58.8–79.5%)	92.9% (90.0–95.2%)
*ERBB2* (HER2)**** in ER−	27	1	3	48	79	90.0% (73.5–97.9%)	98.0% (89.1–99.9%)	89.1% (78.7–99.5%)	94.9% (87.5–98.6%)
*MKI67* (Ki67)^xxxx^	117	63	15	94	289	88.6% (82.0%–93.5%)	59.9% (51.8%–67.6%)	47.1% (37.6–56.6%)	73% (67.5–78%)
*MKi67* (Ki67)^xxxxx^	155	25	37	72	289	80.7% (74.5–85.7%)	74.2% (64.7–81.9%)	53.3% (43.2–63.5%)	78.6% (73.4–82.9%)

*Using the IHC cut-off of 1% as recommended by ASCO-CAP (2010)

^x^Using the IHC cut-off of 10% cut-off, as described elsewhere

**Using central IHC and central FISH for resolution of IHC 2+ to either FISH-negative or FISH-positive, as recommended by the ASCO-CAP guidelines for HER2 testing (2013/2014)

^xx^Comparison of STRAT4 *ERBB2* d*C*_t_ result and HER2 result with IHC 2+ equivocals excluded from analysis using the Herceptest

***In the ER-positive subset only (as determined by IHC), overall percent agreement (OPA), positive percent agreement (PPA), and negative percent agreement (NPA) are shown for the comparison of STRAT4 *ERBB2* d*C*_t_ result and HER2 result by central IHC and central FISH where IHC 2+ equivocals were resolved to positive or negative calls by the FISH assay

^xxx^Comparison of STRAT4 *ERBB2* d*C*_t_ result and HER2 result by central FISH

****In the ER-negative subset only (as determined by IHC), overall percent agreement (OPA), positive percent agreement (PPA), and negative percent agreement (NPA) are shown for the comparison of STRAT4 *ERBB2* d*C*_t_ result and HER2 result by central IHC and central FISH where IHC 2+ equivocals were resolved to positive or negative calls by the FISH assay

^xxxx^Comparison of STRAT4 *MKi67* d*C*_t_ result and Ki67 result by central IHC using a Ki67 IHC cutoff of 20% to discriminate “high proliferation rate” from “low proliferation rate”

^xxxxx^Comparison of STRAT4 *MKi67* d*C*_t_ result and Ki67 result by central IHC using a Ki67 IHC cutoff of 10% to discriminate “high proliferation rate” from “low proliferation rate”

**Fig. 1:**
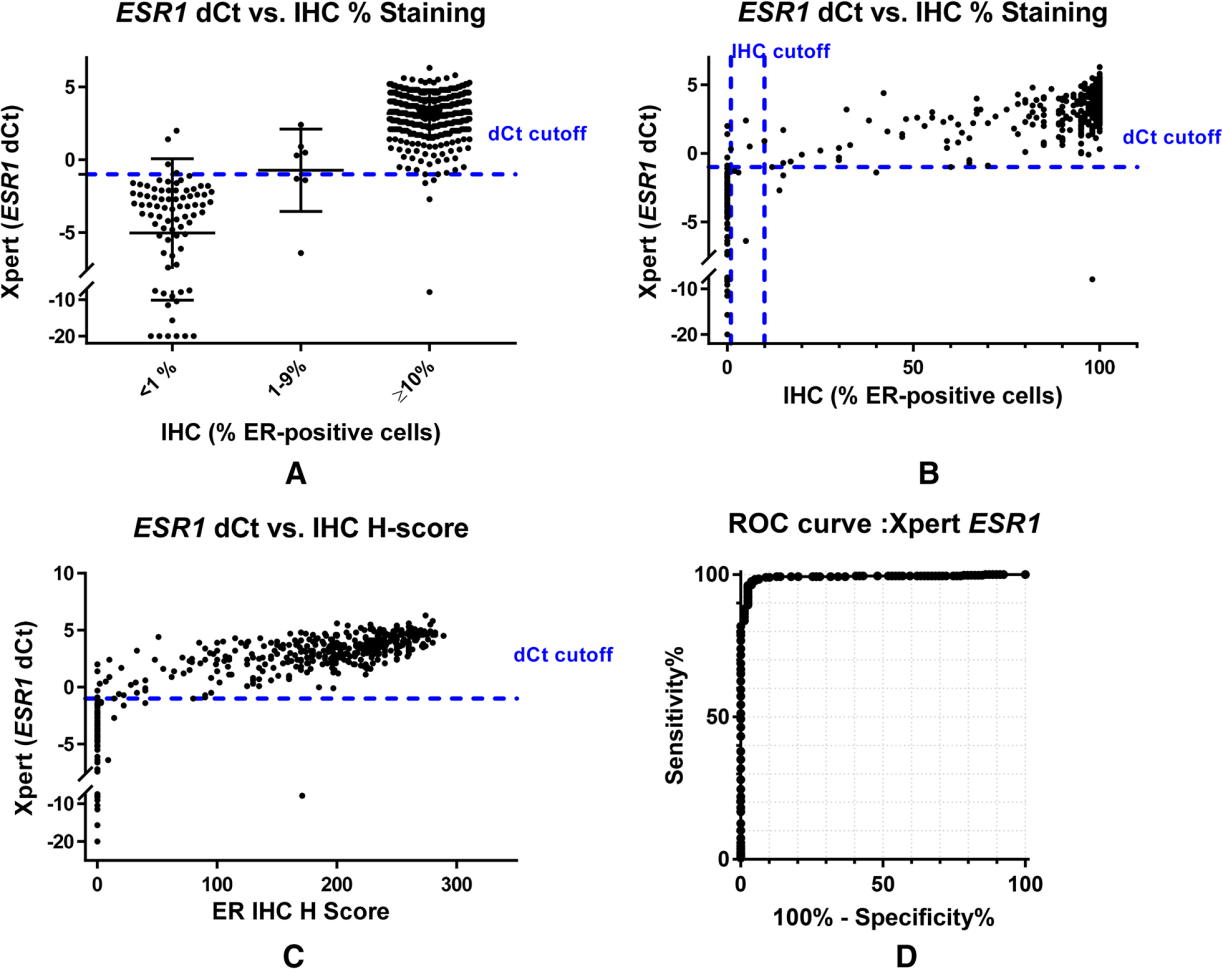
Comparison of estrogen receptor status determined by immunohistochemistry and RT-qPCR (STRAT4 or [STRAT4 (Xpert)]) assays. **a** Graph of STRAT4 *ESR1* d*C*_t_ values by ER IHC result categorized as negative (0%), low positive (1–9%), or positive (≥ 10%). Among ER-positive and ER-negative breast cancers according to IHC assessment, there is also a clear separation by *ESR1* mRNA by RT-qPCR into high and low expression subgroups. In contrast, those breast cancers with from 1 to 9% ER-positive carcinoma cells have predominantly *ESR1* mRNA quantities near the RT-qPCR cut-off separating “positive” from “negative”. **b** Comparison of STRAT4 *ESR1* d*C*_t_ values according to ER IHC % staining alone. The plot of ER IHC percentage positive tumor cell immunohistochemical staining demonstrated a strong correlation with *ESR1* mRNA quantity. **c** Graph of STRAT4 *ESR1* d*C*_t_ values by ER IHC H-Score. H-Score is defined as [3(% of tumor staining 3+)] + [2(% of tumor staining 2+)] + [1(% of tumor staining 1+)]. Quantitative stratification of the IHC protein assessment by combining percentage of immunostained tumor cells with intensity of immunohistochemical staining demonstrated an improved correlation with *ESR1* mRNA determined by RT-qPCR. **d** The ROC curve for STRAT4 *ESR1* including all samples in the analysis. The area under the curve (AUC) is 0.99

*ESR1* d*C*_t_ values were plotted against percent positive staining treated as categorical or continuous variables and H score for the same samples (Fig. 1a–c). These data suggest high levels of concordance between STRAT4 and central IHC for *ESR1*/ER, and demonstrate that the discordant samples are nearly all close to the *ESR1* d*C*_t_ cutoff.

### Agreement rates between *PGR* mRNA and PR protein by IHC

**Fig. 2:**
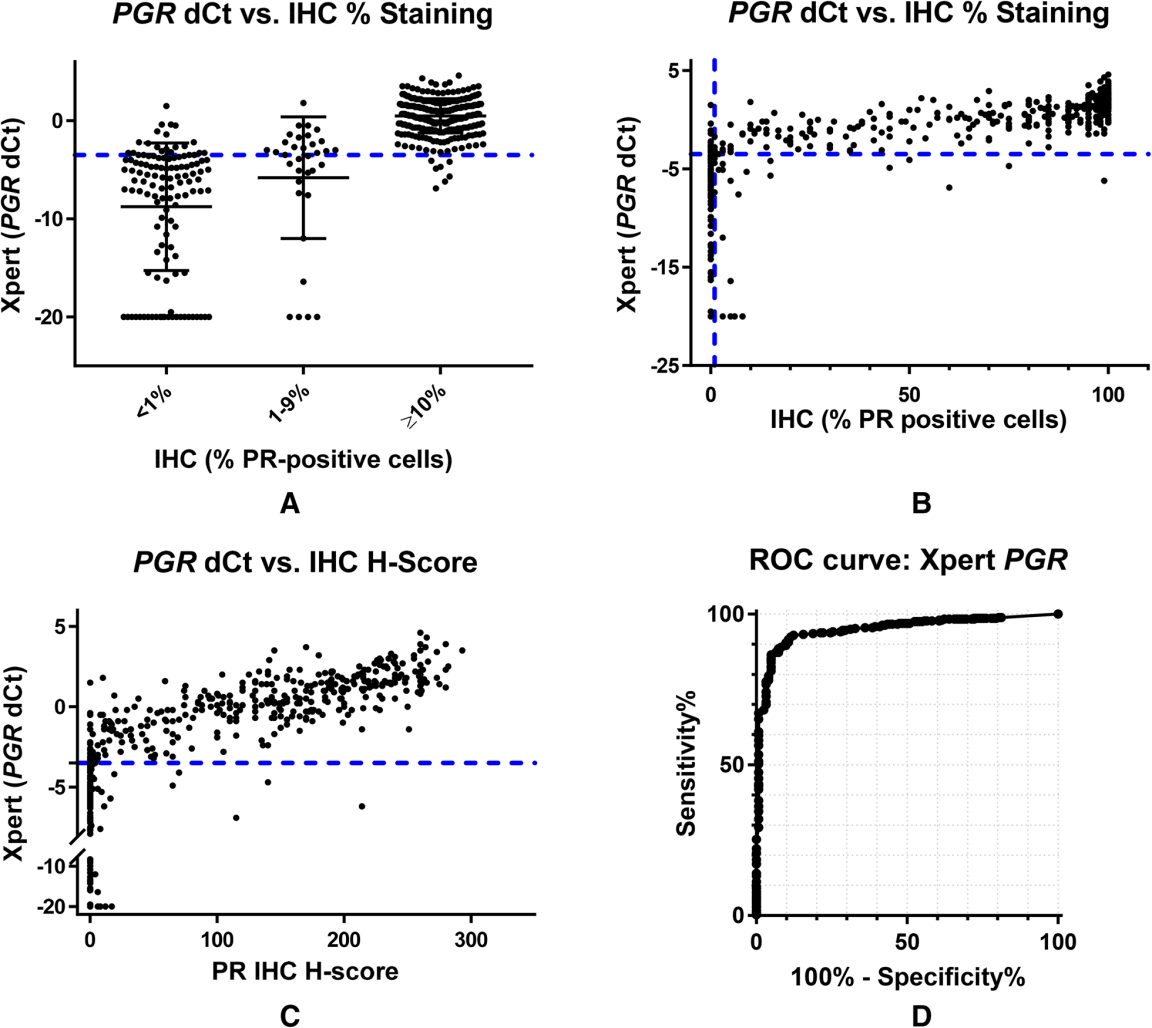
Comparison of progesterone receptor status determined by immunohistochemistry (IHC) and RT-qPCR (STRAT4) assays. **a** Graph of STRAT4 *PGR* d*C*_t_ values by PR IHC result categorized as negative (0%), low positive (1–9%), or positive (≥ 10%). Among PR-positive and PR-negative breast cancers according to IHC assessment, there is also a relatively good separation by *PGR* mRNA by RT-qPCR into high and low expression subgroups. In contrast, the majority of those breast cancers with 1–9% PR-positive carcinoma cells have predominantly *PGR* mRNA quantities near the RT-qPCR cut-off separating “positive” from “negative”. **b** Graph of STRAT4 *PGR* d*C*_t_ values by PR IHC % staining. The plot of the percentage of PR-positive tumor cells by immunohistochemical staining demonstrated a strong correlation with *PGR* mRNA quantity which was improved only slightly by consideration of tumor cell IHC staining intensity as shown in **c. c** Graph of STRAT4 *PGR* d*C*_t_ values by PR IHC H-Score. H-Score is defined as [3(% of tumor staining 3+)] + [2(% of tumor staining 2+)] + [1(% of tumor staining 1+)]. Quantitative stratification of the IHC protein assessment by combining percentage of immunostained tumor cells with intensity of immunohistochemical staining demonstrated a slightly improved correlation with *PGR* mRNA determined by RT-qPCR. **d** The ROC curve for STRAT4 *PGR* including all samples in the analysis. The area under the curve (AUC) is 0.95

The overall concordance rate between the STRAT4 *PGR* results and the central PR IHC results with the PGR 636 antibody was in the 90–91% range whether 1 or 10% staining was used to determine PR-positive status (Table 1). Fifteen samples with “indeterminate” STRAT4 *PGR* results (delta *C*_t_ < − 3.5 and CYFIP *C*_t_ > 31) were excluded from the concordance analysis. The correlation between *PGR* d*C*_t_ values and PR IHC results considered as a categorical variable suggests that there are more samples close to the *PGR* d*C*_t_ cutoff with different IHC staining results, explaining the lower overall percent agreement for *PGR* (Fig. 2a). Of samples considered as low positives by IHC (1–9% PR staining), roughly half were called positive by STRAT4 and half were called negative. Scatterplots of STRAT4 *PGR* d*C*_t_ values compared with PR IHC percent staining and H Score suggest a positive correlation between the absolute level of *PGR* transcript detected and the absolute amount of PR staining observed by IHC (Fig. 2b, c).

### Agreement rates between *ERBB2* mRNA and HER2 protein by IHC (and FISH)

The overall concordance rate between STRAT4 *ERBB2* and HER-2 IHC was 95.7%, excluding samples with staining results of “2+” (Table 1). Concordance between STRAT4 and FISH alone was 91.1% (Table 1). When STRAT4 was compared to IHC plus FISH, where IHC 2+ samples were tested by FISH and categorized as either ISH-positive or ISH-negative, the overall positive agreement rate was 93.3% (Table 1). Finally, similar concordance rates were obtained when the population was stratified first by ER status (Table 1).

**Fig. 3:**
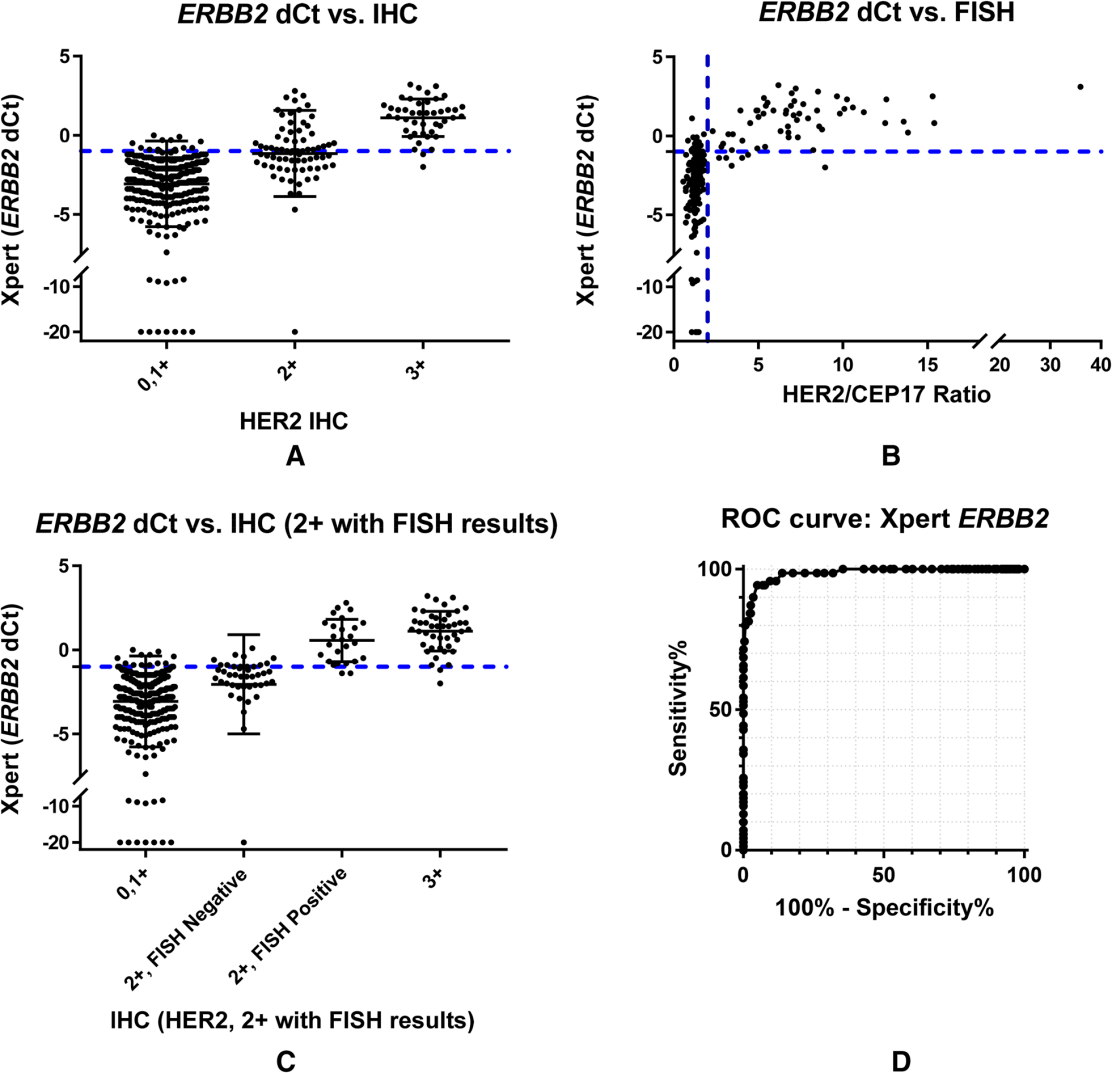
Comparison of human epidermal growth factor receptor 2 determined by either RT-qPCR or by immunohistochemistry with or without FISH assessment of IHC2+. **a** Graph of STRAT4 *ERBB2* d*C*_t_ values by HER2 IHC result categorized as negative (0–1+), equivocal (2+), or positive (3+). There is a significant correlation between HER2 status determined by IHC and *ERBB2* mRNA determined by RT-qPCR with IHC 0/1+ showing low-level expression of *ERBB2* mRNA and IHC 3+ showing high-level *ERBB2* mRNA expression. The IHC 2+ breast cancers appear to have a level of *ERBB2* mRNA intermediate between that of the IHC 0/1+ and IHC 3+ subgroups. **b** Graph of STRAT4 *ERBB2* d*C*_t_ values by HER2/CEP17 Ratio by the FISH assay. Increasing levels of gene amplification determined by FISH (increasing HER2/CEP17 ratio) are associated with increasing levels of *ERBB2* mRNA expression, as expected. **c** Graph of STRAT4 *ERBB2* d*C*_t_ values by IHC plus FISH where FISH was used to resolve the IHC 2+ equivocals into HER2-positive or HER2-negative status. Although the IHC 2+ breast cancers appear to have a level of *ERBB2* mRNA intermediate between that of the IHC 0/1+ and IHC 3+ subgroups (as illustrated in A above), further stratification can be achieved by the use of FISH in this group to determine which cases are *HER2*-amplified and which are not. Those IHC 2+ breast cancers with *HER2*-amplification by FISH have *ERBB2* mRNA expression levels similar to IHC 3+ breast cancers. In contrast, those IHC 2+ breast cancers that are HER2-not-amplified by FISH have *ERBB2* mRNA expression levels similar to IHC 1+ breast cancers. **d** The ROC curve for STRAT4 *ERBB2* including all samples in the analysis. The area under the curve (AUC) is 0.99

Comparison of STRAT4 *ERBB2* d*C*_t_ values and IHC results demonstrated minimal overlap in the IHC negative (0–1+) and IHC positive (3+) populations (Fig. 3a), while the IHC equivocal group (2+) is almost perfectly bisected by the *ERBB2* d*C*_t_ cutoff. A comparison between *ERBB2* d*C*_t_ values and the HER-2/CEP17 ratios determined by FISH demonstrates an apparent correlation between increasing HER-2/CEP17 ratios and the amount of *ERBB2* transcript detected by STRAT4 (Fig. 3b).

Figure 3c shows the correlation between *ERBB2* d*C*_t_ values by STRAT4 and the combined results generated when IHC and FISH are used together to determine HER-2 over-expression. Among those patients who were IHC 2+, STRAT4 for *ERBB2* overwhelmingly agreed with FISH when it came to resolving those patients into HER-2 positive and HER-2 negative groups.

Stratifying the population into ER-positive and ER-negative sub-populations and then examining the correlation between the *ERBB2* d*C*_t_ values and HER-2 results by IHC + FISH suggests that the assay is slightly better at discriminating over-expressors in the ER-negative than the ER-positive sub-population, but the difference is small (Supplementary Fig. 1).

### Agreement rates between *MKi67* mRNA and Ki67 proliferation rates determined by IHC

**Fig. 4:**
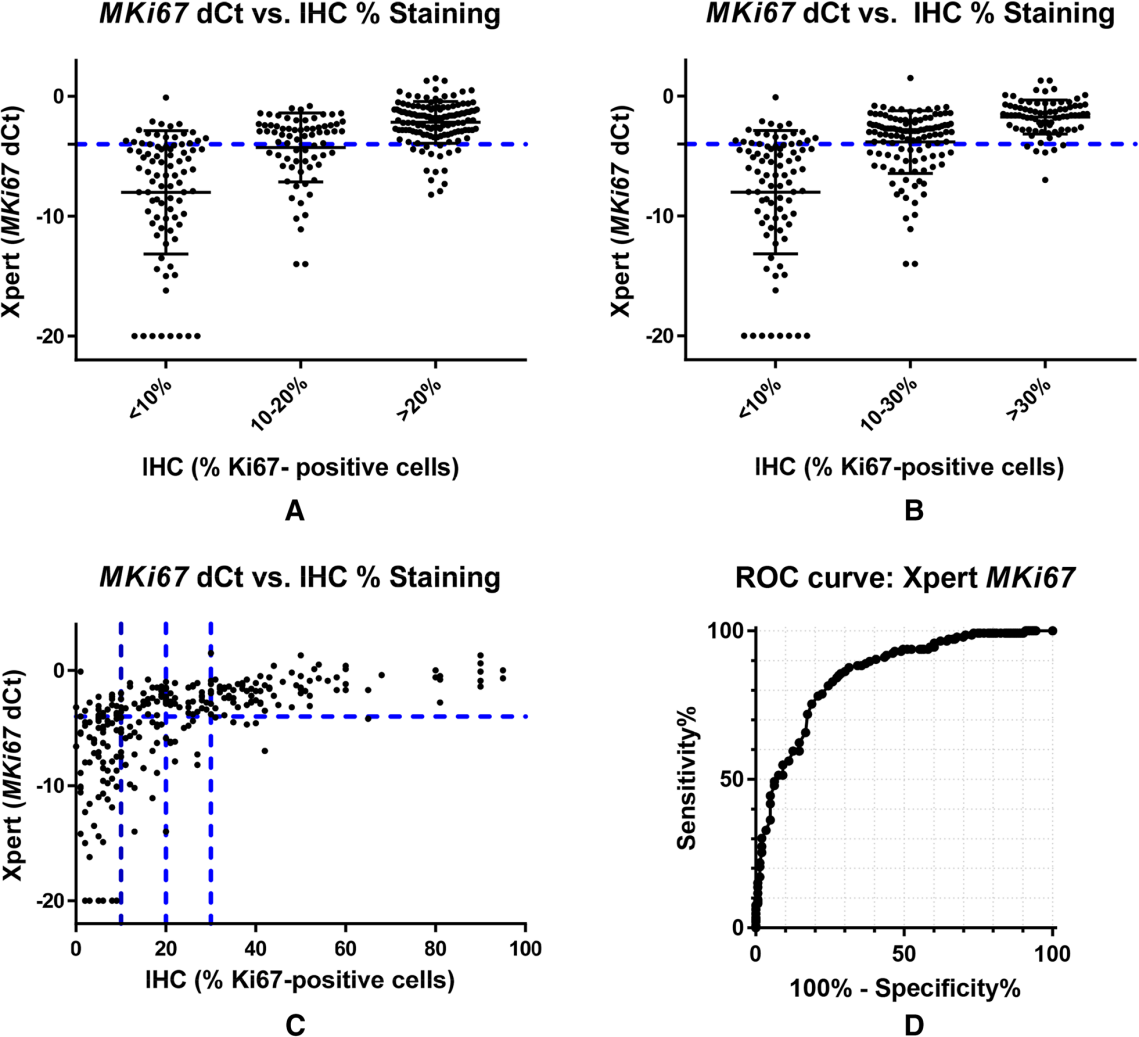
Comparison of Ki67 proliferation rate determined by either RT-qPCR or immunohistochemistry. **a** Graph of STRAT4 *MKi67* d*C*_t_ values by Ki67 IHC % staining where the IHC high proliferation rate cutoff is defined as 20% and the intermediate proliferation rate is defined as 10–20% with < 10% considered a low proliferation rate. There is some overlap in *MKi67* mRNA values between the high (> 20%) and low (< 10%) proliferation rate groups, with the intermediate group (10–20%) showing intermediate mRNA values by RT-qPCR, but with substantial overlap with both the high and low proliferation rate groups. *MKi67* as measured by RT-qPCR appears as a continuum without a clear cutoff evident from the distributions when compared to Ki67 levels measured by IHC. **b** Graph of STRAT4 *MKi67* d*C*_t_ values by Ki67 IHC % staining where the IHC positivity cutoff is defined as 30% and the equivocal zone is defined as 10–30%. Raising the IHC cutoff for the determination of high proliferation rate has no appreciable impact on the correlation between RT-qPCR and IHC methods. The *MKi67* distribution still shows a continuum of values without a clear cutoff. **c** Graph of STRAT4 *MKi67* d*C*_t_ values by Ki67 IHC % staining. There appears to be a discernable correlation between the percentage of tumor cells with immunochemical staining for Ki67 with *MKi67* mRNA levels by RT-qPCR, especially at levels above 40%. **d** The ROC curve for STRAT4 *MKi67* where all samples were included in the analysis. The area under the curve (AUC) is 0.85

For *MKi67*/Ki67 concordance, twenty-four samples with indeterminate STRAT4 results were excluded from the analysis. We examined the correlation between *MKi67* by RT-qPCR and Ki67 by IHC using cutoffs of 10% and 20% for the determination of high proliferation rate. Although many laboratories consider < 10% to indicate low proliferation rate, > 20% to indicate high proliferation rate, and use an intermediate zone between 10 and 20%, we elected to choose single cutoffs for these analyses because we had not previously defined an intermediate zone for the *MKi67* d*C*_t_ distribution. The overall positive agreement rate for STRAT4 *MKi67* results with Ki67 by IHC using a 20% cutoff was 73%, and using a 10% cutoff was 78.6%. (Table 1). Comparison of *MKi67* d*C*_t_ values with Ki67 IHC results categorized as low (< 10%), intermediate (10–20%), or high (> 20%) offers a possible explanation for these lower concordance rates. Rather than distinct subsets, the distribution appears as a continuum with the large intermediate population showing substantial overlap with both the low and high populations, although the median values of each sub-population are clearly different, and correlate positively with increasing *MKi67* d*C*_t_ values by STRAT4 (Fig. 4a). Expanding the intermediate zone to include patients with Ki67 IHC values of 10–30% leads to the same conclusion (Fig. 4b). Comparison of *MKi67* d*C*_t_ values with a continuous measure of IHC % immunostaining for Ki67 demonstrates a weak correlation with STRAT4 and significant scatter at low proliferation rates below 30% (Fig. 4c).

### Receiver-operator characteristic analyses

STRAT4 results were compared with central IHC/FISH using receiver-operator characteristic (ROC) analyses for all four analytes (Figs. 1d, 2d, 3d, 4d). The ROC area under the curve (AUC) values for each of the four analytes were 0.99 for *ESR1* and *ERBB2*, 0.95 for PGR, and 0.85 for MKi67. For *MKi67*, the AUC value improved to 0.92 when the equivocal samples (IHC Ki67 staining of 10–20%) were excluded from the analysis (Supplementary Fig. 2). The shape of the curves for *ESR1* and *ERBB2*, the two most important analytes for LMIC use, demonstrated that the STRAT4 assay is highly correlated with results generated using conventional IHC and FISH assays. While the *PGR* assay results were highly correlated according to the ROC curve, they were not as highly correlated as *ESR1* and *ERBB2*. The ROC curve for *MKi67* STRAT4 and conventional IHC was the weakest of the four correlations. Whether all patients were included in the analysis, or the 10–20% intermediate group were excluded, the data suggest that it is more difficult to identify a clear cutoff when the distribution appears to define a continuum, an observation that has been suggested even when using IHC to measure this analyte [19].

### Variability of STRAT4 concordance with different IHC antibodies and scoring methods

The antibodies selected for the primary concordance analysis in this study were the antibodies used at the central reference laboratory, but not all reference laboratories used the same antibodies to perform IHC for these four analytes. In order to get a sense of how disparate the concordance between STRAT4 and IHC might be when compared across a variety of central reference laboratories, we selected a subset of samples (*n* = 155) and tested them at three different central reference laboratories that used different IHC antibodies and scoring methods. The concordance rates for STRAT4 *ESR1* to IHC ER range from 97.8% for the 6F11 antibody scored manually to 91.9% for the SP1 antibody using an automated scoring system (Supplementary Fig. 3). Concordance across different IHC methods for ER were in the 94–95% range (data not shown).

For STRAT4 *PGR* to PR IHC comparisons, the concordance ranged from 94.4 to 89% while the IHC to IHC comparisons were similar at 94.3 and 93.6% (Supplementary Fig. 4).

For the STRAT4 *ERBB2* to IHC HER2 comparisons, with equivocal breast cancers (IHC 2+ samples) excluded, concordance ranged from 94.3 to 92.8%, and from 93.3 to 91.6% if FISH was employed to resolve the IHC 2+ samples (Supplementary Table 1). IHC to IHC comparisons using different antibodies were not performed.

For the STRAT4 *MKi67* to IHC Ki67 comparisons, excluding samples with IHC staining in the 10–20% range, concordance was quite variable depending on the antibody/method comparator. Using the MIB1 antibody and manual scoring, concordance was 84.6%, while the same antibody used in a different central laboratory and employing an automated scoring system yielded only a 63.7% concordance. Another automated scoring system using antibody 30-9 was intermediately concordant at 76%. A comparison of the STRAT4 *MKi67* d*C*_t_ values to % staining with these three antibodies/methods is shown in Supplementary Fig. 5.

## Discussion

The measurement of mRNAs that encode protein biomarkers like ER, PR, HER2, and Ki67 (*ESR1, PGR, ERBB2*, and *MKi67*, respectively) on an automated, broadly distributed diagnostic platform is feasible and carries certain advantages for LMIC applications, including ease-of-use, accessibility, standardization, reproducibility, and a short time-to-result. These features suggest that such an approach has real potential for clinical benefit in the management of patients with breast cancer in low- and middle-income countries, where accessing more standard diagnostic methods like IHC and FISH is difficult. The ASCO/CAP guidelines currently recommend IHC and/or FISH for the determination of hormone receptor or HER-2 status for the purpose of selecting therapy, therefore, it is important that we determine the degree of concordance between STRAT4 and these standard methods [12–14].

In prior studies, we have shown that STRAT4 is analytically valid and capable of generating reproducible results even when variability in pre-analytical sample handling exists across laboratories performing the test. Our goal in this study was to determine the degree of concordance between measurements of the mRNAs *ESR1, PGR, ERBB2*, and *MKi67* by STRAT4 and central laboratory IHC and FISH results for ER, PR, HER2, and Ki67.

The results demonstrate high concordance for *ESR1*/ER and *ERBB2*/HER2 with ROC AUC values of 0.99 for both, and very good concordance for *PGR*/PR (ROC AUC = 0.95). For *MKi67*/Ki67, including all samples in the analysis, the ROC AUC was reduced to 0.85. This may not be surprising, however, due to the significant challenges already described in achieving standardization of Ki67 result reporting by IHC across laboratories [15–18]. Denkert and colleagues [19] have shown that Ki67 results by IHC are highly variable unless at least 500, and better yet 1000, cells are carefully counted for each sample. Multicenter studies to address improved methods of standardization for Ki67 measurement by IHC are in progress. For these markers, examination of direct correlations between the RNA measurements and clinical outcomes is desirable, but this is particularly important for Ki67, where the establishment of *MKi67* d*C*_t_ cutoffs derived directly from clinical outcomes would obviate the need for further concordance studies and perhaps offer pathologists an easier method of reliably measuring this proliferation marker.

The results of the antibody comparison studies demonstrate some minor variabilities in concordance rates depending on the laboratory, antibody, and scoring method used for *ESR1*/ER, *PGR*/PR, and *ERBB2*/HER2, suggesting that the STRAT4 assay results are generally concordant across central laboratories using different methods. For *MKi67*/Ki67, there was significant variability in concordance, but once again, this is not unexpected given the challenges associated with Ki67 assessments by IHC.

The d*C*_t_ cutoffs used for all four of the STRAT4 analytes were pre-specified based on prior testing in small datasets. However, we learned during the performance of this study that reliable negative results for both *PGR* and *MKi67* depended upon having a larger amount of sample available for analysis by the cartridge than it did for *ESR1* or *ERBB2*. Based on these observations, the requirement was established that for each of these two analytes, the *CYFIP1* internal control *C*_t_ value needed to be ≤ 31 rather than 35, which is the *CYFIP1 C*_t_ cutoff for *ESR1* and *ERBB2*. Thus, select samples were classified as indeterminate if the *PGR* or *MKi67* values were below the cutoff for positivity and also had a *CYFIP1 C*_t_ of greater than 31. Moving forward, such samples would be re-run using the lysate from the entire FFPE section rather than only 520 µL, which represents 25% of the final lysate volume.

The current study suggests that the determination of *ESR1, PGR, ERBB2*, and *MKi67* RNA levels by RT-qPCR on the GeneXpert automated diagnostic platform is not only feasible, but also generates results from FFPE tumor sections that are highly concordant with high quality central laboratory measurements of ER, PR, and HER2 using standardized IHC and FISH assays. For Ki67, the continuous nature of the distribution of values we observed as well as known difficulties in standardizing IHC assessments across laboratories presents unique challenges, but one we hope to address more fully by using clinical outcomes from appropriately designed studies to define the STRAT4 *MKi67* d*C*_t_ cutoff(s) that will provide the clearest and most clinically informative interpretation.

STRAT4 has already been shown to be highly concordant with automated quantitative analysis of IHC (AQUA) [20]. From a LMIC perspective, *ESR1* and *ERBB2* measurements have the greatest relevance currently. As such, given these results, the STRAT4 assay could be considered a potential solution to the problem of limited access to breast cancer diagnostics that currently exists for patients in low resource countries, and should move forward to a prospective concordance study without delay, paying particular attention to the impact of local sample handling and fixation methods on STRAT4 results. Assuming the STRAT4 assay can be validated for use in those geographies, prior experience with the GeneXpert system can be leveraged for rapid progress toward a workable diagnostic solution for patients with breast cancer in countries with limited healthcare resources, particularly LMIC.

## Electronic supplementary material

Below is the link to the electronic supplementary material.

Supplementary material 1 (DOCX 807 KB)
