# Supplementary material for: Comparison of central laboratory assessments of ER, PR, HER2, and Ki67 by IHC/FISH and the corresponding mRNAs (ESR1, PGR, ERBB2, and MKi67) by RT-qPCR on an automated, broadly deployed diagnostic platform
Source: Breast Cancer Res Treat. 2018 Aug 17;172(2):327–38. doi: 10.1007/s10549-018-4889-5 (PMC6208911; doi:10.1007/s10549-018-4889-5)
Supplement: Supplementary file 1 — Supplementary material 1 (DOCX 807 KB) [file 10549_2018_4889_MOESM1_ESM.docx]

**Supplemental Data**

**Supplemental Table 1. Comparison of *ERBB2* mRNA by RT-qPCR with Protein Status for HER2 by IHC using different anti-HER2 Antibodies and Platforms**.

| Analyte | IHC+/ RTqPCR+ | IHC-/ RTqPCR+ | IHC+/ RTqPCR- | IHC-/ RTqPCR- | Total Number | Sensitivity (PPA) (95% CI) | Specificity (NPA) (95% CI) | Kappa Statistic (95% CI) | Concordance Rate (OPA) (95% CI) |  |
| --- | --- | --- | --- | --- | --- | --- | --- | --- | --- | --- |
| *ERBB2* (HER2)* | 9 | 5 | 1 | 90 | 105 | 90.0% (55.5 – 99.7%) | 94.7% (88.1% - 98.3%) | 71.9 (50.7% - 93%) | 94.3% (88.0 – 97.9%) |  |
| *ERBB2* (HER2)^xx^ | 11 | 7 | 2 | 105 | 125 | 84.6% (54.6 – 98.1%) | 93.8% (87.5% - 97.5%) | 67% (47% - 86.9%) | 92.8% (86.8 – 96.7%) |  |
| *ERBB2* (HER2)^xxx^ | 10 | 8 | 3 | 110 | 131 | 76.9% (46.2 – 95.0%) | 93.2% (87.1% - 97.0%) | 59.9% (38.5% - 81.2%) | 91.6% (85.5 – 95.7%) |  |
| *ERBB2* (HER2)**** | 11 | 7 | 2 | 115 | 135 | 84.6% (54.6 – 98.1%) | 94.3% (88.5 – 97.7%) | 67.3% (47.5% - 87.1%) | 93.3% (87.7 – 96.9%) |  |
|  |  |  |  |  |  |  |  |  |  |  |
|  |  |  |  |  |  |  |  |  |  |  |

*Comparison of STRAT4 *ERBB2* dCt result and HER2 result by central IHC using the Herceptest scored manually and with exclusion of equivocal (IHC 2+) breast cancers.

^xx^Comparison of STRAT4 *ERBB2* dCt result and HER2 result by central IHC using the 4B5 antibody, scored using automation and with exclusion of equivocal (IHC 2+) breast cancers.

^xxx^Comparison of STRAT4 *ERBB2* dCt result and HER2 result by central IHC using the Herceptest and FISH to resolve the status of the IHC 2+ breast cancers.

****Comparison of STRAT4 *ERBB2* dCt result and HER2 result by central IHC using the 4B5 antibody and FISH to resolve the status of the IHC 2+ breast cancers.

**Supplemental Figures**

Supplemental Figure 1. Stratifying the cohort into ER-positive and ER-negative subsets, graph of STRAT4 (Xpert) *ERBB2* dCt values by IHC plus FISH where FISH was used to resolve the IHC 2+ equivocals into positive or negative calls. While there is an obvious correlation between IHC/FISH negative and positive status and *ERBB2* mRNA levels by RT-qPCR, there is more overlap in the positive and negative distributions in the ER-positive sub-population than in the ER-negative subpopulation.

|  |  |
| --- | --- |
|  |  |
|  |  |

Supplemental Figure 2. The ROC curve for STRAT4 (Xpert) *MKi67* where the IHC high proliferation rate cutoff was set at 20% and samples with Ki67 IHC staining in the 10-20% range were excluded from the analysis. Exclusion of patients with Ki67 IHC values in the 10-20% range improves the correlation between IHC and RT-qPCR for *MKi67*. The area under the curve (AUC) is 0.92.

|  |  |
| --- | --- |
|  |  |
|  |  |

Supplemental Figure 3. Scatterplot of STRAT4 (Xpert) *ESR1* dCt values by multiple methods of IHC using different antibodies and scoring methods. While there are differences in the distributions based on the IHC antibody and method used for assessment, there is an overall good correlation between *ESR1* by RT-qPCR and ER by IHC regardless of the comparator method used with OPA ranging from 91.9% to 97.8%.

|  |  |
| --- | --- |
|  |  |
|  |  |

Supplemental Figure 4. Scatterplot of STRAT4 (Xpert) *PGR* dCt values by multiple methods of IHC using different antibodies and scoring methods. While there are differences in the distributions based on the IHC antibody and method used for assessment, there is an overall good correlation between *PGR* by RT-qPCR and PR by IHC regardless of the comparator method used with OPA ranging from 89% to 94.4%.

|  |  |
| --- | --- |
|  |  |
|  |  |

Supplemental Figure 5. Scatterplot of STRAT4 (Xpert) *MKi67* dCt values by multiple methods of IHC using different antibodies and scoring methods. The antibody and the method employed to assess Ki67 proliferation rate had an impact on the degree of correlation with *MKi67* by RT-qPCR, with OPA ranging from 63.7% to 84.6%.

|  |  |
| --- | --- |
|  |  |
|  |  |
